# Dual-Target *Mycobacterium tuberculosis* Inhibition: Insights into the Molecular Mechanism of Antifolate Drugs

**DOI:** 10.3390/ijms241814021

**Published:** 2023-09-13

**Authors:** Pritika Ramharack, Elliasu Y. Salifu, Clement Agoni

**Affiliations:** 1 Biomedical Research and Innovation Platform (BRIP), South African Medical Research Council (SAMRC), Cape Town 7505, South Africa; 2 Discipline of Pharmaceutical Sciences, School of Health Sciences, Westville Campus, University of KwaZulu-Natal, Durban 4001, South Africa; 3 UCD Conway Institute of Biomolecular and Biomedical Research, University College Dublin, D04 V1W8 Belfield, Ireland

**Keywords:** *Mtb* drug resistance, dual-target modulation, molecular dynamic simulations, cheminformatics

## Abstract

The escalating prevalence of drug-resistant strains of *Mycobacterium tuberculosis* has posed a significant challenge to global efforts in combating tuberculosis. To address this issue, innovative therapeutic strategies are required that target essential biochemical pathways while minimizing the potential for resistance development. The concept of dual targeting has gained prominence in drug discovery against resistance bacteria. Dual targeting recognizes the complexity of cellular processes and disrupts more than one vital pathway, simultaneously. By inhibiting more than one essential process required for bacterial growth and survival, the chances of developing resistance are substantially reduced. A previously reported study investigated the dual-targeting potential of a series of novel compounds against the folate pathway in *Mycobacterium tuberculosis*. Expanding on this study, we investigated the predictive pharmacokinetic profiling and the structural mechanism of inhibition of UCP1172, UCP1175, and UCP1063 on key enzymes, dihydrofolate reductase (DHFR) and 5-amino-6-ribitylamino-2,4(1*H*,3*H*)-pyrimidinedione 5′-phosphate reductase (RV2671), involved in the folate pathway. Our findings indicate that the compounds demonstrate lipophilic physiochemical properties that promote gastrointestinal absorption, and may also inhibit the drug-metabolizing enzyme, cytochrome P450 3A4, thus enhancing their biological half-life. Furthermore, key catalytic residues (Serine, Threonine, and Aspartate), conserved in both enzymes, were found to participate in vital molecular interactions with UCP1172, which demonstrated the most favorable free binding energies to both DHFR and RV2671 (−41.63 kcal/mol, −48.04 kcal/mol, respectively). The presence of characteristic loop shifts, which are similar in both enzymes, also indicates a common inhibitory mechanism by UCP1172. This elucidation advances the understanding of UCP1172’s dual inhibition mechanism against *Mycobacterium tuberculosis.*

## 1. Introduction

Over the past decade, tuberculosis (TB) has become the leading cause of death globally from an infectious disease, as reported by the World Health Organization [1]. The infectious agent responsible for TB, *Mycobacterium tuberculosis* (*Mtb*), has a unique ability to survive within a host by alternating between active and latent disease states while evading the host’s immune system defenses. Despite research efforts, the discovery of effective tuberculosis therapies has been insufficient, as more than 10 million people are affected each year, as reported by Furin et al. (2019) [2]. Although first- and second-line treatment regimens are currently available against resilient bacteria, several strains of *Mtb* have become resistant to drugs such as isoniazid, rifampicin, and ciprofloxacin, leading to extensively drug-resistant TB [3]. A newly developed drug approved by the FDA, pretomanid, is currently available for combinative use with bedaquiline and linezolid in the treatment of highly resistant TB. Although the drug is effective against resistant strains of the bacteria, it has been associated with numerous adverse effects, including peripheral neuropathy and hyperamylasemia, as reported by Conradie et al. (2022) [4]. 

In recent years, mutant strains of *Mtb* have emerged, leading to amino acid alterations within the bacterial proteins, resulting in increased and/or complete resistance to therapeutic regimens. Given these concerns, researchers have been exploring new techniques, including dual-target modulation therapeutics. Key benefits of these dual-target treatments are the use of a single drug against two targets in a metabolic pathway, as well as increased drug-repurposing potential. However, there are several viewpoints regarding dual-target modulation, including concerns regarding toxicological effects and adverse drug reactions, as well as the potential for drug “promiscuity” [5].

Examples of dual-target inhibitors were identified in a study carried out by Hajian et al. (2019) [6]. The study identified a series of ionized non-classical antifolates (INCAs) that exhibited antibacterial properties in the *Mtb* folate pathway. Of the INCAs, UCP1172 demonstrated the most favorable antimicrobial and biochemical characteristics, followed by UCP1175 and UCP1063. These compounds have the ability to target two rate-limiting enzymes, dihydrofolate reductase (DHFR) and 5-amino-6-ribitylamino-2,4(1*H*,3*H*)-pyrimidinedione 5′-phosphate reductase (RV2671), involved in the folate pathway [6]. Other known dual inhibitors that target key enzymes in the folate metabolic pathway include Methotrexate (MTX) [7], Pemetrexed [8], Raltitrexed [9], Tomudex [10], and Trimetrexate [11]. These have all been identified to simultaneously inhibit both dihydrofolate reductase (DHFR) and thymidylate synthase (TS), which are key enzymes involved in nucleotide synthesis and DNA replication [12].

The folate metabolic pathway is essential for the production of folate, which is an essential cofactor required for the synthesis of methionine, *N*-formylmethionyl-tRNA, glycine, serine, pantothenate, purines, thymidine, and deoxythymidine monophosphate (dTMP) [13]. One of the crucial enzymes in the folate pathway is DHFR, which is encoded by the folA gene and required to catalyze the reduction of dihydrofolate (DHF) to tetrahydrofolate (THF) [14]. The 5-amino-6-ribitylamino-2,4(1*H*,3*H*)-pyrimidinedione 5′-phosphate (AROPP) reductase enzyme, also known as RV2671, was previously thought to be solely involved in the riboflavin biosynthetic pathway. However, further studies, including a report by Cheng et al. (2017), have shown that the enzyme can reduce DHF to THF, similar to the mechanism of DHFR [15]

**Figure 1:**
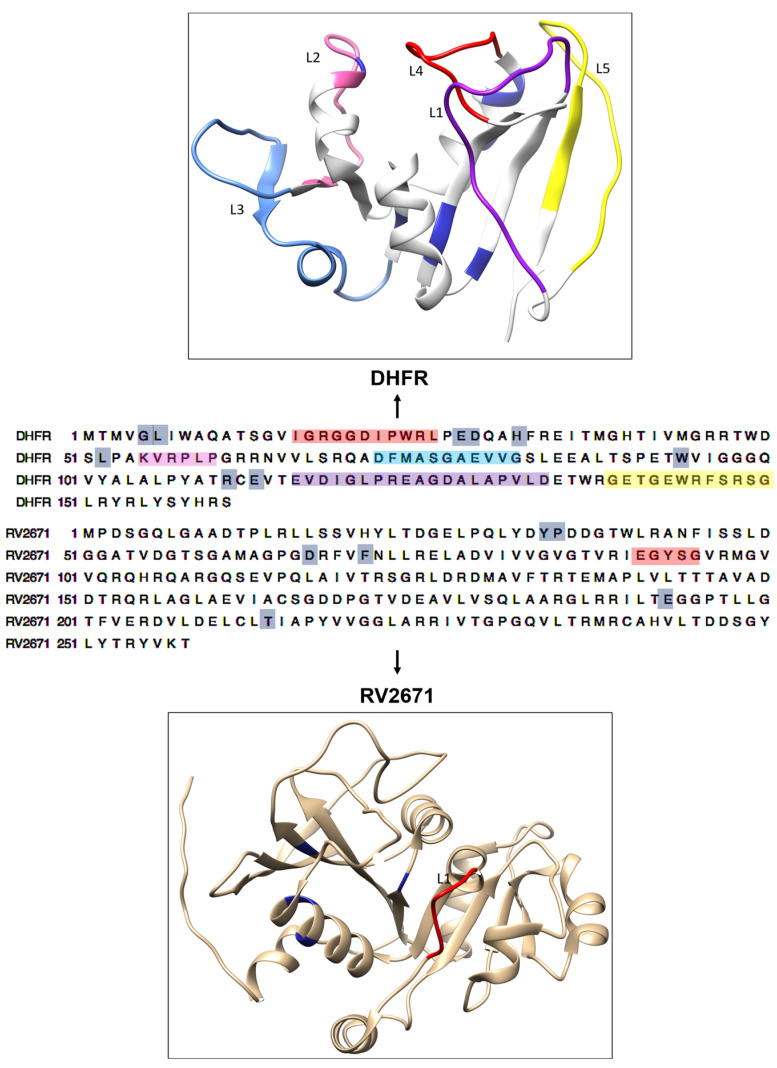
The overall structure and sequence analysis of *Mtb* DHFR and RV2671 enzymes. The DHFR structure above shows five loops (L1 (residues 116–134) in purple, L2 (residues 55–60) in pink, L3 (residues 72–82) in light blue, L4 (residues 16–26) in red, and L5 (residues 139–150) in yellow). The RV2671 structure below DHFR consists of a β-strand and α-helices with a characteristic L1 loop (residues 91–95: depicted in red), which is similar to the L2 loop in DHFR. Active site residues of both enzymes are shown in blue.

A study by Dias et al. (2014) expanded upon the work of Bystroff et al. (1991) and Sawaya et al. (1997) by describing the structural characteristics of *Mtb* DHFR during the catalytic cycle of the folate pathway [16,17,18]. The DHFR enzyme is composed of an adenosine domain and a loop domain that contains the characteristic *Mtb* L1, L4, and L5 loops. The L2 and L3 loops are small, rigid regions located at the adenosine domain. The L1 loop located in RV2671, plays a significant role in its function and interaction with other molecules. This loop is a dynamic region that contributes to the protein’s overall structure and function. The L1 loop’s conformational changes are often associated with key functional transitions, such as direct binding with co-factor, NADP^+^. These changes might be vital for the enzymatic function of RV2671 [15] (see Figure 1).

In order to gain insights into the structural and molecular mechanisms underlying the dual inhibition of DHFR and RV2671, we conducted an investigation using pharmacokinetic profiling and molecular dynamics simulations on INCAs, UCP1172, UCP1175, and UCP1063. This study aimed to further elucidate the shared molecular mechanism of these dual inhibitors within *Mtb’s* folate pathway.

## 2. Results 

### 2.1. Predicting the Pharmacokinetic Properties of the Dual-Inhibitor INCAs

UCP1172, UCP1175, and UCP1063 demonstrated the most favorable antimicrobial and biochemical characteristics amongst those produced by Hajian et al. (2019) [6]. However, adverse physiological effects are a major concern with most clinical anti-tuberculosis drugs. Therefore, it is important to assess the metabolic efficacy and toxicological profile of the compounds by measuring their pharmacokinetic properties [19]. To determine the compounds’ drug-like nature and predict their pharmacokinetic properties, the physicochemical descriptors of the compounds were computed using the online software SWISSADME, as presented in Table 1. SWISSADME utilizes the BOILED-Egg method, which computes the lipophilicity and polarity of small molecules [20]. 

As observed in Table 1, all the compounds comply with Lipinski’s rule of 5, indicating that the molecules may be suitable anti-tuberculosis drug leads. The calculated lipophilicity of the compounds ranged from 3.06 to 3.18, indicating a hydrophobic nature. Although the INCAs have a high gastrointestinal absorption rate, the molecules were not predicted to cross the blood–brain barrier due to their large molecular weights (373.45–416.47 g/mol). The SWISSADME analysis also predicted all three compounds to inhibit the CYP P450 3A4 enzyme, whilst UCP1172 and UCP1175 inhibit P-gp, which, taken together, indicate increased biological half-lives of the molecules [21]. By inhibiting cytochrome P450 3A4, it is expected that the molecules are not metabolized in the liver and thus directly target DHFR and RV2671. The synthetic accessibility scores of the molecules were found to be between 3.40 and 3.96, indicating easy chemical synthesis (1 = easily synthesized, while 10 = difficult) [22]. Based on these physiochemical properties, oral administration of these molecules is predicted to have favorable pharmacokinetic characteristics. However, further experimental evidence is needed to support these hypotheses.

Pan-assay interference compounds (PAINS) are chemical substances that often result in false-positive outcomes during high-throughput screening by non-specifically reacting with various biological targets instead of specifically affecting the intended target [23]. Fortunately, all three molecules were not classified as PAIN compounds, indicating that they may not generate false positives in high-throughput screening experiments.

### 2.2. Defining the Structural Integrity of the INCAs’ Antibacterial Potency

**Figure 2:**
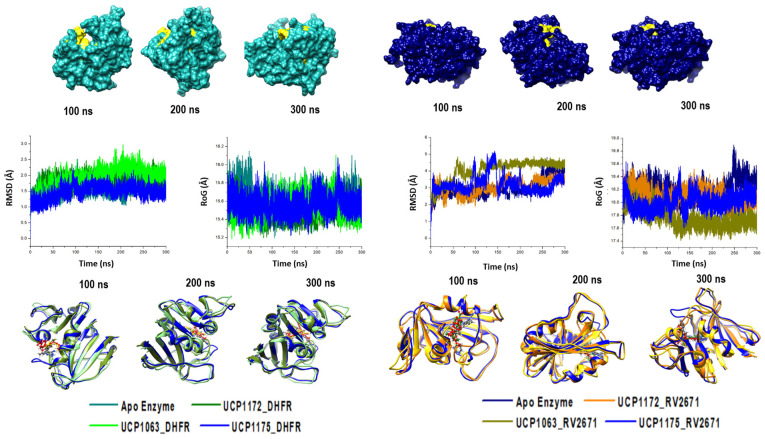
The RMSD and RoG profiles of the UCP1172, UCP1175, and UCP1063 bound to DHFR and RV2671 complexes were compared to those of APO DHFR (in cyan) and RV2671 (in navy). It can be observed that binding of the inhibitors on the DHFR enzyme do not have a significant impact on the structural dynamics during the 300 ns simulation. Major stabilization of the systems was noted upon inhibitor binding in the RV2671 systems.

In order to determine the impact of UCP1172, UCP1175, and UCP1063’s binding on the structural stability of the DHFR and RV2671 enzymes, the root mean square deviation (RMSD) and radius of gyration (RoG) of the alpha-carbons were measured. The APO DHFR and RV2671 enzymes were used as simulated controls to differentiate any differences in the structures. The results of the RMSD and RoG analysis for the systems are shown in Figure 2.

It was evident from the results that UCP1172 dramatically stabilized RV2671 (RMSD: APO—2.98 Å; UCP1172-bound—3.10 Å,) after reaching convergence at approximately 40 ns. A similar effect was observed in the bound systems of UCP1175 and UCP1063 (UCP1063-bound—4.03 Å and UCP1175-bound—3.19 Å). In contrast, all DHFR systems maintained minimal displacement throughout the trajectory (RMSD: APO—1.59 Å; UCP1172-bound—1.80 Å, UCP1063-bound—1.95 Å, and UCP1175-bound—1.50 Å). 

To further highlight the reliability of the simulated systems, the RMSD of the active site residues on both enzymes, as well as the stability of UCP1172 (most optimal experimental inhibitor), was determined (Figures S1 and S2). The findings revealed stable active sites, with RMSD averages for RV2671 (APO—3.62 Å; bound—2.78 Å) and DHFR (APO—2.44 Å; bound—2.40 Å).

A structural visualization via snapshots from the simulation further revealed UCP1172 within the active site of DHFR and RV2671 engaged in a unidirectional movement as shown in Figure S2. It was observed that throughout the 300 ns simulation, UCP1172 maintained steady positioning with minimal displacement from its rotational center, further indicating the stability of UCP1172 at the active sites of DHFR and RV2671.

Complimentary results were noted in the compactness of the systems. In the RV2671 systems, all bound inhibitors condensed the atomistic dispersion observed in the APO enzyme, whereas no suggestive fluctuations were noted in the DHFR systems. The dynamic modifications observed in RV2671 could be a result of simulating a monomeric unit of the enzyme, suggesting a dimeric structure is necessary for the rigidity of the enzyme.

**Figure 3:**
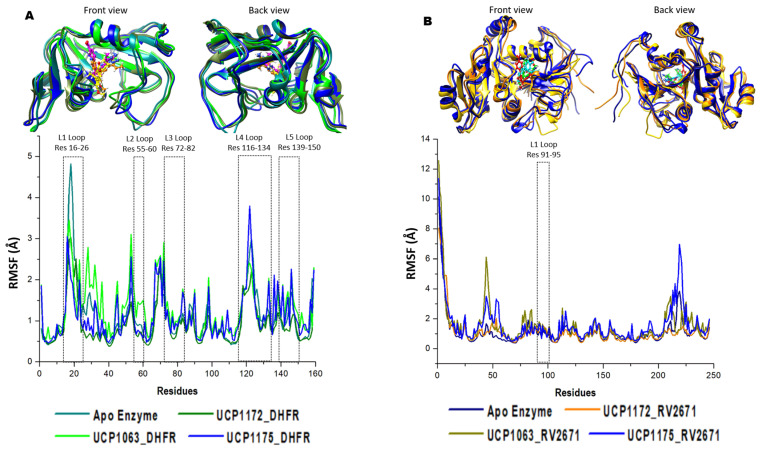
RMSF graphical analysis of the (**A**) UCP1172, UCP1175, and UCP1063 bound to DHFR (olive, blue, green, APO enzyme is depicted in cyan) and (**B**) UCP1172, UCP1175, and UCP1063 bound to RV2671 (orange, blue, dark yellow, APO enzyme is depicted in navy) complexes. Highly fluctuating residues are noted in the focused domains of the image.

To analyze the residual evolution of the systems during the trajectory, the root mean square fluctuation (RMSF) of each amino acid was assessed (Figure 3).

As a result of the 300 ns simulation, residual fluctuations of both enzymes were noted subsequent to INCA binding. It was interesting to note that key loops involved in the structural activity of DHFR enzyme displayed major fluctuations through the simulation. In L2, L3, L4, and L5 (location displayed in Figure 1), all bound systems displayed higher fluctuations during the simulation when compared to the APO enzyme. This indicated key dynamic shifts in the loops surrounding the active site. Interesting, L1, which is a loop that was exposed to the substrate, the APO complex displayed much higher fluctuations when compared with the bound systems. It was also observed that the UCP1063 complex demonstrated more fluctuations between L1 and L2, whilst the UCP1175 complex demonstrated higher DHFR fluctuations within the L4–L5 loop. Although RV2671 demonstrates a variable loop between residues 91–95, all systems demonstrated a uniform fluctuation within this area. It was interesting to note that between residues 35 and 55 and 215 and 225, fluctuations were noted in the UCP1063 and UCP1175 complexes, respectively.

To measure the binding landscape of UCP1172 to DHFR in comparison to RV2671, thermodynamic free-binding energy calculations, per-residue decomposition, and ligand-interaction plot analysis were carried out.

### 2.3. Deciphering the Commonality between the Active Site of DHFR and RV2671

**Figure 4:**
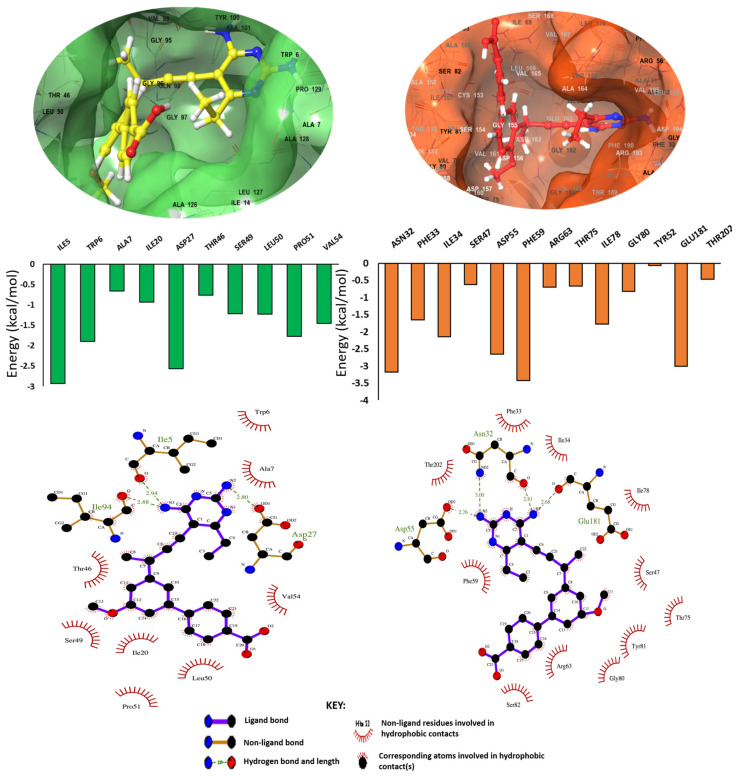
Binding landscape and molecular interaction plots of the DHFR and RV2671 complexes. Graphs demonstrated the energy contributions of each active site residue to the final free-binding energy of each system.

The Molecular Mechanics/Generalized Born Surface Area (MM/GBSA) method was employed to estimate the binding free energetics of the UCP1172 bound complexes. The free energy calculations were carried out for UCP1175 and UCP1063 bound systems as well. The MM/GBSA method has become an established approach for predicting binding energy; it is well known to be more accurate than most molecular docking scoring functions and computationally less strenuous than most alchemical free energy approaches [24]. Molecular dynamic free-binding energy calculations consider the molecular forces that contribute to the interactions between the compound and the protein. This final force takes into account the energy of the protein and compound by itself, as well as the final delta energy of the complex. Table 2 describes the free-binding energy of the enzyme, UCP1172, and final delta energy (ΔG_bind_) in each system. Table 2 further highlights the energy terms that contribute to the binding free energy, while Figure 4 depicts the binding landscape analysis for DHFR and RV2671. 

It was noted that although UCP1172 contributed similar energies in both systems, RV2671 demonstrated greater energy contributions in comparison to DHFR. This was due to augmented van der Waals and electrostatic energy within the enzyme’s structure, thus correlating with the instability seen in the structural analysis of the APO enzyme. This increase in RV2671’s energy led to a greater final energy of the complex (−48.03 kcal/mol) compared to DHFR’s final complex (−41.63 kcal/mol). In the UCP1175 and UCP1063 bound systems, the binding free energies between each inhibitor against the DHFR and RV2671 were similar as well, with major contributions from the van der Waals and electrostatic components. However, UCP1172 significantly showed the most favorable binding to the two targets when all three INCAs were compared. To establish the residues contributing to the binding of UCP1172 to the drug pockets, ligand interactions plots were assessed. Clear hydrogen bond interactions were noted between the terminal amine groups of UCP1172 and ILE5, ASP27, and ILE94 of DHFR, as well as ASN32, ASP55, and GLU181 of RV2671. The results of residues contributing to the binding of UCP1175 and UCP1063 are highlighted in the Figures S4 and S5. Common hydrogen bonds were noted for residues ILE5, ASP27, and ILE94 for the UCP117-DHFR complex, whilst conserved hydrogen bonds with ASP27 were noted for the UCP1063-DHFR complex. It was interesting to note that for the RV2671 complexes, conserved hydrogen bonds with ASN32 and GLU181 were noted between UCP1172 and UCP1063. However, there was no observed hydrogen bond conservation between UCP1175 and the other INCA complexes.

In summary, UCP1172 demonstrated the most optimally bound INCA to both DHFR and RV2671; this was corroborated by the best free-binding energy results and favorable molecular interactions when complexed with *Mtb* enzymes.

## 3. Discussion

In this study, we investigated the molecular mechanism of action of UCP1172 against the *Mtb* folate pathway through the dual inhibition of DHFR and RV2761. This was subsequently corroborated by the evaluation of two other INCAs, UCP1175 and UCP1063, both of which exhibited significant inhibitory effects on both enzymes.

A drug’s pharmacokinetic profile is critical in determining its physiological effectiveness and safety. For drugs intended for human use, a balance between pharmacokinetics, potency, and selectivity is crucial. Human oral bioavailability is an essential pharmacokinetic parameter, and the LogP value, which affects a compound’s solubility, permeability, or hepatic clearance, should ideally be between 2 and 3 [25]. Pharmacokinetic profiling indicated favorable drug-like characteristics for all three molecules. This was attributed to their high GIT absorption, facilitated by the optimal lipophilic nature of the compounds. This observation was corroborated by Hajian et al. (2016), who reported similar partition coefficient (LOGP) values for the molecules [26]. The noteworthy lipophilicity of these compounds enables enhanced permeation through the *Mtb* cell wall, subsequently increasing the likelihood of reaching the target enzymes. 

Furthermore, the inhibitory effect on the metabolizing enzyme cytochrome P450 3A4 was observed for all three molecules. This suggests that these molecules are not metabolized by this enzyme, consequently leading to an extended biological half-life. Additionally, it was observed that both UCP1172 and UCP1175 do not function as substrates for P-gp. In contrast, UCP1063 demonstrated substrate behavior for P-gp, implying its potential for excretion through this mechanism in comparison to the other two molecules. This discrepancy could be attributed to the lower molecular weight of UCP1063 or the pyridine terminal end of the molecule. However, it is worth noting that there is a lack of previous experimental evidence to validate these findings. 

When compared with UCP1172, both UCP1175 and UCP1063 demonstrated structural stabilization of both the folate pathway enzymes, being DHFR and RV2671. Characteristic loop modifications accompanied this to accommodate the compound at the active site of each enzyme. Interestingly, both enzymes produced similar structural modifications upon INCA-binding, indicating a similar inhibitory mechanism of the compound. This may be due to attraction forces between molecular groups of the compound and the amino acids constituting the loop. This has been previously evidenced by Dias et al. (2014), who noted that upon binding of a natural substrate, the L1 and L2 loops close over the cavity, holding the substrate at the active site [18]. Cheng et al. (2017) described the RV2671 homodimer which contains an active site to which the natural substrate, NADP+, binds. Upon this binding at the C-terminal domain of the enzyme, a disordered segment (residues 91–95) forms a loop, however, no other significant structural modifications are noted. Interestingly, the above-mentioned loop corresponds to that of the L2 loop located in DHFR, thus suggesting similar functional roles of the enzymes [15]. It was interesting to note that similar interactions were demonstrated upon binding of UCP1175 and UCP1063, thus validating the structural modifications of these enzymes upon INCA binding. To further validate the “closing” of the cavity over the active site and the inhibitory effects which were noted, Hajian et al. (2019) assessed the recovery rate constant from a jump dilution assay. When bound with the inhibitors, a natural substrate was used to assess the competitive inhibition. It was noted that UCP1172 (0.024), followed by UCP1175 (0.072) and UCP1063 (0.096), demonstrated similar recovery rates to the positive control, methotrexate (0.056) [6]. This indicates the amount of inhibitor that was recovered subsequent to dilution with the natural substrate.

An explanation of the conformational shifts could be explained by the permeation of the active site pocket by UCP1172. Molecular dynamic simulations allow for the compound to form molecular interactions with the active site residues. By this dynamic virtue, amino acids shift during a trajectory generated in the simulation, thus allowing the compound to become energetically stable within the available pocket. The greater the complementarity of the compound with the active site residues, the longer it will remain within the binding pocket [27]. This provides a blockade against dihydrofolate binding, thus halting conversion to tetrahydrofolate. This also further validates the stabilizing effect of UCP1172 on the inconsistent fluctuations observed in the RV2671 APO enzyme. 

The structural analysis revealed the compounds’ confinement within the active site. Empirical evidence indicated UCP1172’s stronger binding, contrasting with other INCAs and the positive control. This was supported by free energy binding calculations over a 300 ns simulation, consistently showing UCP1172’s superior binding energies compared to UCP1175 and UCP1063. This was validated by the shared binding sites used by the molecules within their respective enzyme contexts.

Favorable molecular interaction patterns supported these findings, with similar residues surrounding the active site and influencing the binding energy. Notably, hydrogen and hydrophobic bonds between residues of both enzymes and UCP1172 were remarkably alike. Despite distinct numerical designations, the characteristic ASP residue was present in both DHFR and RV2671 systems. Common active site residues in both DHFR and RV2671 complexes included hydrophobic interactions from THR and SER, impacting the free-binding energy in both systems. These residues are known as essential catalytic elements in crystallographic studies with inhibitors [6,28,29], further confirming UCP1172’s inhibitory activity in both enzyme complexes. 

These “double-edged” inhibitors have the potential to maintain effective anti-TB therapy in mutant strains. We believe that this study not only provides insights into effective TB therapy but also helps prevent the emergence of new drug resistant *Mtb* strains. Moreover, understanding the mechanistic role of these inhibitors within the folate pathway could lead to potential treatments against a plethora of diseases.

## 4. Material and Methods

### 4.1. Measurement of Pharmacokinetic Properties and Drug Likeliness of INCAs

The SWISSADME server was used to determine the physicochemical descriptors, pharmacokinetic properties, and drug-like nature of UCP1172, UCP1175, and UCP1063. The “Brain Or Intestinal Estimated permeation (BOILED-Egg)” method was utilized as it computes the lipophilicity and polarity of small molecules [22].

### 4.2. DHFR System Preparation

The X-ray crystal structure of the DHFR-UCP1172 (PDB code: 6DDP) and DHFR-UCP1175 (PDB code: 6DDS) complexes were obtained from the RSCB Protein Data Bank [30]. Using the UCSF Chimera software package (Version 1.15) [31], the DHFR enzyme was prepared by removing the ligand, repeated monomers, and any other non-essential substrates, including water. The UCP1172 compound was prepared on the Avogadro software package (Version 1.2) [32], where the molecular geometry of the compound was optimized, and hydrogen atoms added as per requirements for molecular dynamic simulations. 

Due to there being no available crystal structure of UCP1063 with DHFR, molecular docking of the compound at the active site of DHFR was carried out. The active site residues of DHFR were obtained from the DHFR-UCP1172 complex and the optimized UCP1063 compound from PDB code 6DE5 was utilized for docking. The Molecular docking software employed in this study utilized the Autodock Vina Plugin (Version 1.2.0) available on Chimera [33] with default docking parameters. Prior to docking, Gasteiger charges were added to UCP1063 and the non-polar hydrogen atoms were merged to carbon atoms. The molecule was then docked into the binding pocket of DHFR (by defining the grid box with a center of −2.94 × 5.34 × 4.61 and size of 13 × 14 × 13, pointing in *x*, *y*, and *z* directions). The enzyme and compound were subsequently separated and prepared as per the UCP1172-DHFR system. 

### 4.3. RV2671 System Preparation

The X-ray crystal structure of the RV2671-UCP1063 system (PDB code: 6DE5) was obtained from the RSCB Protein Data Bank [30]. The RV2671 enzyme was then prepared as per DHFR on the UCSF Chimera software package (Version 1.15) [31]. The UCP1063 compound was prepared on the Avogadro software package (Version 1.2) [31] where the molecular geometry of the compound was optimized, and hydrogen atoms were added as per requirements for molecular dynamic simulations.

Due to there being no available crystal structure of UCP1172 and UCP1175 with RV2671, molecular docking of the compounds at the active site of RV2671 was carried out. The active site residues of RV2671 were obtained from PDB code 6DE5 and the optimized UCP1172 and UCP1175 compound from the DHFR system was utilized for docking. The Molecular docking software employed in this study utilized the Autodock Vina Plugin (Version 1.2.0) available on Chimera [33] with default docking parameters. Prior to docking, Gasteiger charges were added to UCP1172 and the non-polar hydrogen atoms were merged to carbon atoms. The molecule was then docked into the binding pocket of RV2671 (by defining the grid box with a center of (−16) × (−24) × 23 and size of 14 × 12 × 12, pointing in *x*, *y*, and *z* directions). The enzyme and compound were subsequently separated and prepared as per the DHFR system. 

For validation purposes, molecular docking of UCP1172 at the active site of RV2671 was conducted by re-docking the native G8J ligand at the active site and then superimposing the two docked complexes. The result showed similarity in binding poses between the two complexes as shown in Figure S3.

### 4.4. Molecular Dynamic Simulations

The molecular dynamic (MD) simulations were performed using the GPU version of the PMEMD engine provided with the AMBER package in which the FF18SB variant of the AMBER force field [34] was used to describe the systems. Molecular dynamic simulations were employed on a total of four systems, inclusive of the APO DHFR and RV2671 enzymes, as well the INCA-DHFR and INCA-RV2671 complexes. 

ANTECHAMBER was used to generate atomic partial charges for the ligand by utilizing the Restrained Electrostatic Potential (RESP) and the General Amber Force Field (GAFF) procedures. The Leap module of AMBER 18 allowed for the addition of hydrogen atoms, as well as counter ions for the neutralization of all systems. The four systems were then suspended implicitly within an orthorhombic box of TIP3P water molecules such that all atoms were within 8 Å of any box edge.

An initial minimization of 3000 steps was carried out with an applied restraint potential of 10 kcal/mol for both enzymes. An additional full minimization of 1000 steps were further carried out by a conjugate gradient algorithm without restraints.

A gradual heating MD simulation from 0 K to 300 K was executed for 50 ps, such that the systems maintained a fixed number of atoms and volume. The solutes within the systems were imposed with a potential harmonic restraint of 10 kcal/mol and collision frequency of 1 ps. Following heating, an equilibration estimating 500 ps of each system was conducted with the operating temperature being kept constant at 300 K. Additional features, including the pressure, were also kept constant, thus mimicking an isobaric–isothermal ensemble (NPT). The system’s pressure was maintained at 1 bar using the Berendsen barostat. 

The total simulation trajectory was 300 ns. In each simulation, the SHAKE algorithm was employed to optimize the hydrogen atom bonds. The step size of each simulation was 2 fs and an SPFP precision model was used. The simulations coincided with the isobaric–isothermal ensemble (NPT) with randomized seeding, the constant pressure of 1 bar maintained by the Berendsen barostat, a pressure-coupling constant of 2 ps, a temperature of 300 K, and a Langevin thermostat with a collision frequency of 1 ps. All simulations were carried out in triplicate (*n* = 3) with the averages represented in the below results.

### 4.5. Post-Dynamic Simulation and Data Analysis

The coordinates of the four systems were then saved and the trajectories were analyzed every 1 ps using PTRAJ, followed by the analysis of RMSD, RMSF, and Radius of Gyration using the CPPTRAJ module employed in the AMBER 18 suit. Free-binding energy calculations were then carried out on the complexed systems as per AMBER package requirements and in-house protocol optimization [35,36,37]. All raw data plots were then generated using the Origin data analysis software (Version 6.0) [38] and 3D images were created using the UCSF Chimera software package (Version 1.15) [31].

#### Binding Free Energy Analysis via MM/GBSA Method

The Molecular Mechanics/Generalized Born Surface Area (MM/GBSA) [39,40,41,42] method was employed in estimating the binding free energy for each of the INCA-DHFR and INCA-RV2671 complexes. Binding free energy was estimated after 300,000 frames (300 ns) where the systems we identified were stable.

Mathematically, the binding free energy (ΔG_bind_) is calculated as follows:

ΔG_bind_ = G_complex_ − G_receptor_ − G_ligand_ (1)

ΔG_bind_ = E_gas_ + ΔG_sol_ − TS, (2)

where ΔG_bind_ is considered to be the summation of the gas phase and solvation energy terms less the entropy (TS) term.

E_gas_ = E_int_ + E_vdw_ + E_elec_ (3)

E_gas_ is the sum of the AMBER force field internal energy terms E_int_ (bond, angle, and torsion), the covalent van der Waals (E_vdw_), and the non-bonded electrostatic energy component (E_elec_). The solvation energy is calculated from the following equation:

G_sol_ = G_GB_ + G_non-polar_ (4)

G_non-polar_ = γSASA + b (5)

where ΔG_bind_ is taken to be the sum of the gas phase and solvation energy terms less the entropy (TΔS) term and G_complex_ represents energy of the receptor ligand complex, while G_receptor_ and G_ligand_ represent energies of receptor and ligand, respectively. Egas denotes gas-phase energy; Eint signifies internal energy, and E_ele_ and E_vdw_ indicate the electrostatic and van der Waals contributions, respectively. E_gas_ is the gas phase, elevated directly from the FF18SB force terms. G_sol_ denotes solvation free energy and can be decomposed into polar and nonpolar contribution states. The polar solvation contribution, G_GB_, is determined by solving the GB equation, whereas G_SA_, the nonpolar solvation contribution, is estimated from the solvent accessible surface area (SASA) determined using a water probe radius of 1.4 Å. T and S correspond to temperature and total solute entropy, respectively. γ is the coefficient of the surface tension of the solvent, whilst *b* is the fitting parameter, whose values are 0.0054 kcal/mol/Å^2^ and 0.916 kcal/mol, respectively [43]. Per-residue decomposition analyses were also carried out via the MM/GBSA method to estimate individual energy contribution of residues of UCP1172-DHFR and UCP1172-RV2671 complex structures.

## 5. Conclusions

This study investigated the structural and molecular mechanism of inhibition of the dual-target compound UCP1172 on the folate pathway enzymes DHFR and RV2671. This was compared and validated with two other experimentally favorable dual inhibitors, UCP1175 and UCP1063. From the results, it was evident that UCP1172’s structural mechanism is to compete with the natural substrate of the folate pathway and bind to the active site, of which the L1 and L2 loops of the enzymes enclose the molecule. Taken together with previously published experimental assays, it can be noted that UCP1172 has a greater affinity to both DHFR and RV2671 when compared to their natural substrates. By allowing for key interactions between the terminal nitrogen atoms of the molecule, key intermolecular interactions are formed between common residues ASP, THR, and SER. These are noted on both enzymes, thus offering a common molecular mechanism of the molecule. Similar interactions are noted with the less potent inhibitors UCP1175 and UCP1063. In summary, this study provides valuable insights into the inhibitory mechanism of UCP1172 on DHFR and RV2671 shedding light on the structural and molecular interactions underlying its dual-target inhibition. We believe that the identification of the structural mechanism of inhibition in the folate pathway could assist in providing insights into effective TB therapy against various strains of *Mtb*.

## Figures and Tables

**Table 1:** Predictive pharmacokinetic profiling of UCP1172, UCP1175, and UCP1063 using the SWISSADME online tool.

ADME Profile	UCP1172	UCP1175	UCP1063
Drug likeness (Lipinski)	Yes	Yes	Yes
Molecular weight (g/mol)	416.47	402.45	373.45
Lipophilicity (iLOGP)	3.18	3.06	3.10
Water soluble	Soluble	Moderately Soluble	Moderately Soluble
GIT absorption	High	High	High
BBB permeability	No	No	No
CYP P450 3A4 inhibitor	Yes	Yes	Yes
P-gp substrate	No	No	Yes
Synthetic accessibility	3.96	3.40	3.76
PAINS	No	No	No

BBB—blood–brain barrier, GIT—gastrointestinal tract, P-gp—P-glycoprotein, CYP P450 3A4—Cytochrome P450 3A4, PAINS—pan-assay interference structures.

**Table 2:** Binding free energy estimation of DHFR and RV2671 complex systems.

System	Energy Components (kcal/mol)				
	${\Delta E}_{vdw}$	${\Delta E}_{ele}$	${\Delta G}_{gas}$	${\Delta G}_{sol}$	${\Delta G}_{bind}$
UCP1172-DHFR	−51.43 ± 0.37	−21.91 ± 0.68	−73.34 ± 0.64	31.71 ± 0.51	−41.63 ± 0.46
UCP1172-RV2671	−50.93 ± 0.61	−35.94 ± 1.09	−86.86 ± 1.27	38.81 ± 0.94	−48.04 ± 0.78
UCP1063-DHFR	−41.06 ± 0.37	−36.27 ± 1.08	−77.33 ± 0.93	42.13 ± 0.61	−35.20 ± 0.44
UCP1063-RV2671	−53.45 ± 0.30	−30.93 ± 0.33	−84.37 ± 0.45	40.56 ± 0.20	−43.81 ± 0.37
UCP1175-DHFR	−20.02 ± 0.14	−338.37 ± 1.25	−217.89 ± 0.93	191.50 ± 0.98	−26.39 ± 0.33
UCP1175-RV2671	−37.53 ± 0.27	−89.71 ± 2.04	−127.24 ± 2.07	103.60 ± 1.89	−23.64 ± 0.37

ΔE_ele_ = electrostatic energy; ΔE_vdw_ = van der Waals energy; ΔG_bind_ = total binding free energy; ΔG_sol_ = solvation free energy ΔG_gas_ = gas phase free energy.

## Data Availability

Data are contained within the article and Supplementary Material.

## Supplementary Materials

The supporting information can be downloaded at: https://www.mdpi.com/article/10.3390/ijms241814021/s1.
