# Supplementary material for: Dual-Target Mycobacterium tuberculosis Inhibition: Insights into the Molecular Mechanism of Antifolate Drugs
Source: Int J Mol Sci. 2023 Sep 13;24(18):14021. doi: 10.3390/ijms241814021 (PMC10530724; doi:10.3390/ijms241814021)
Supplement: Supplementary file 1 [file ijms-24-14021-s001.zip › ijms-2524908-supplementary.pdf]

Dual-Target Mycobacterium Tuberculosis Inhibition: Insights into the Molecular Mechanism of Antifolate Drugs

Pritika Ramharack [1,2,*], Elliasu Y. Salifu [1] and Clement Agoni [2,3]

Supplementary Figures

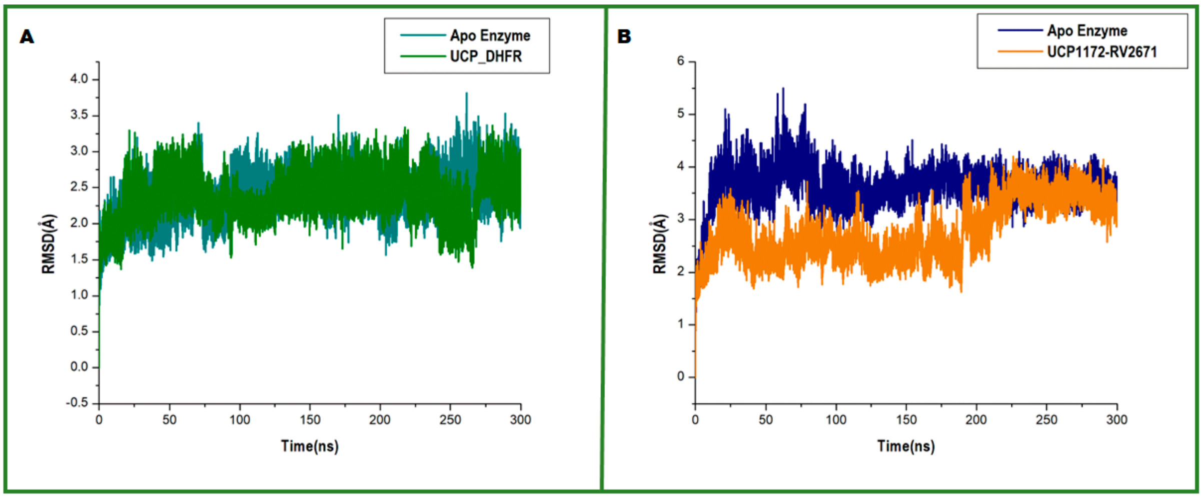

Figure S1: (A) A plot of Cα RMSD showing stability of active site residues in the DHFR systems upon binding of UCP1172 during the 300ns simulation **(B)** A plot of Cα RMSD showing stability of active site residues in the RV2671 systems upon binding of UCP1172 during the 300ns simulation.

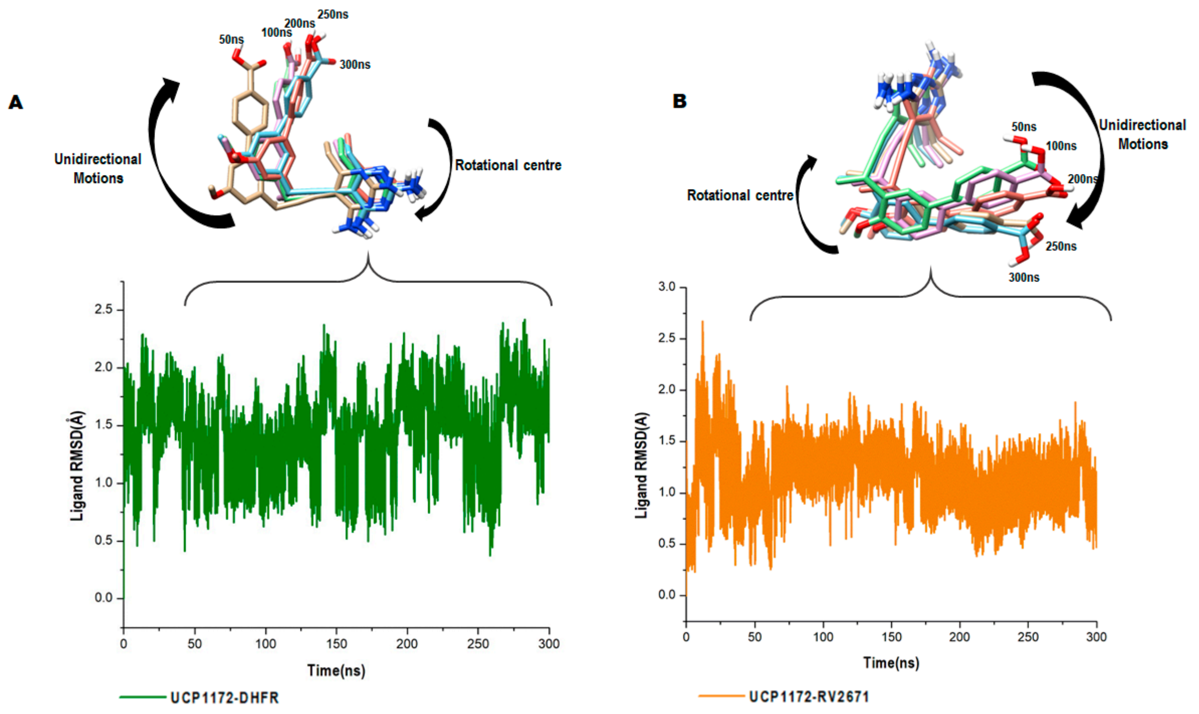

Figure S2: (A) A plot of Cα RMSD showing stability and motions of UCP1172 at the binding site of DHFR and also its differential positioning at selected times over the 300ns simulation **(B)** A plot of Cα RMSD showing stability and motions of UCP1172 at the binding site of RV2671 and also its differential positioning at selected times over the 300ns simulation.

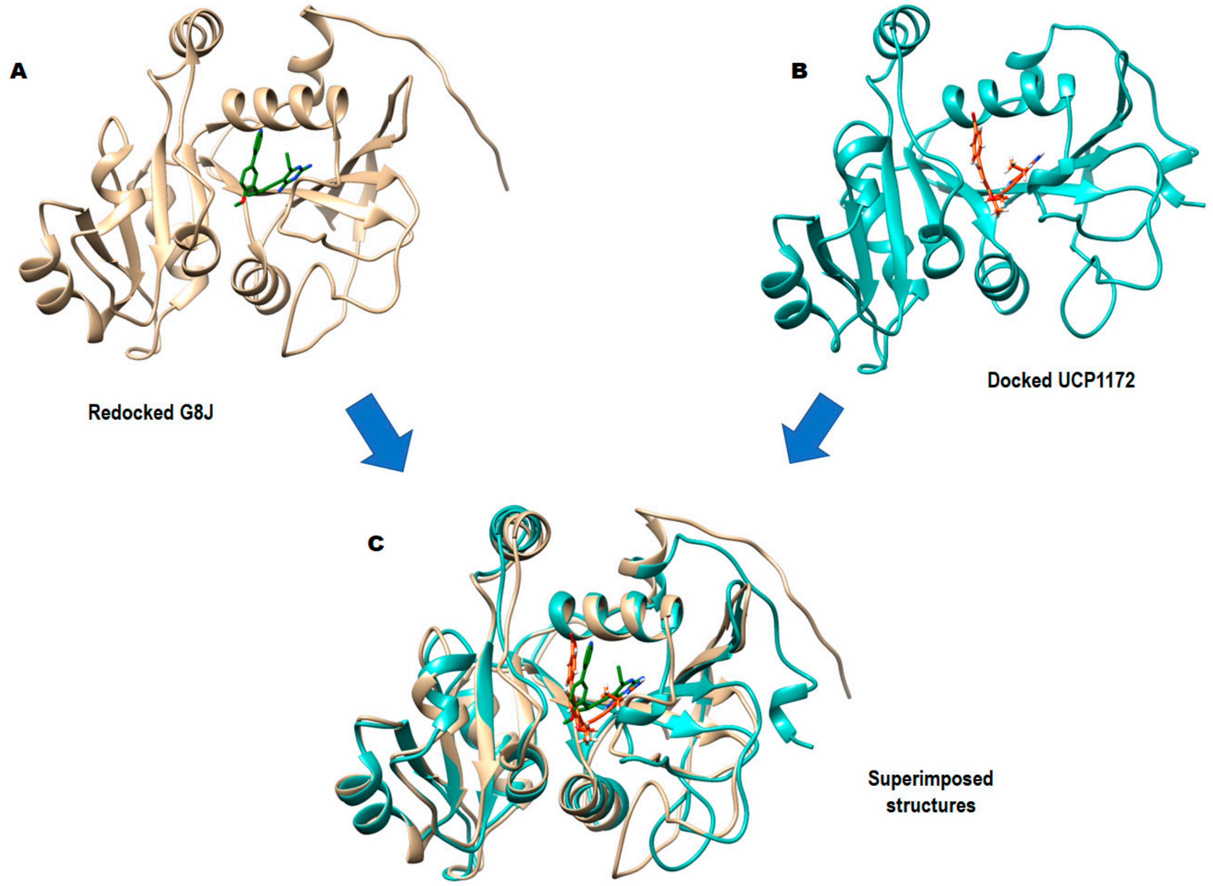

Figure S3: A) Showing the binding pose of the redocked structure of the native ligand G8J at the active site of RV2671 **B)** Showing the best pose of UCP1172 at the binding pocket of RV2671 **C)**Superimposed structures highlighting a similarity in binding poses between the two structures validating the docking protocol.

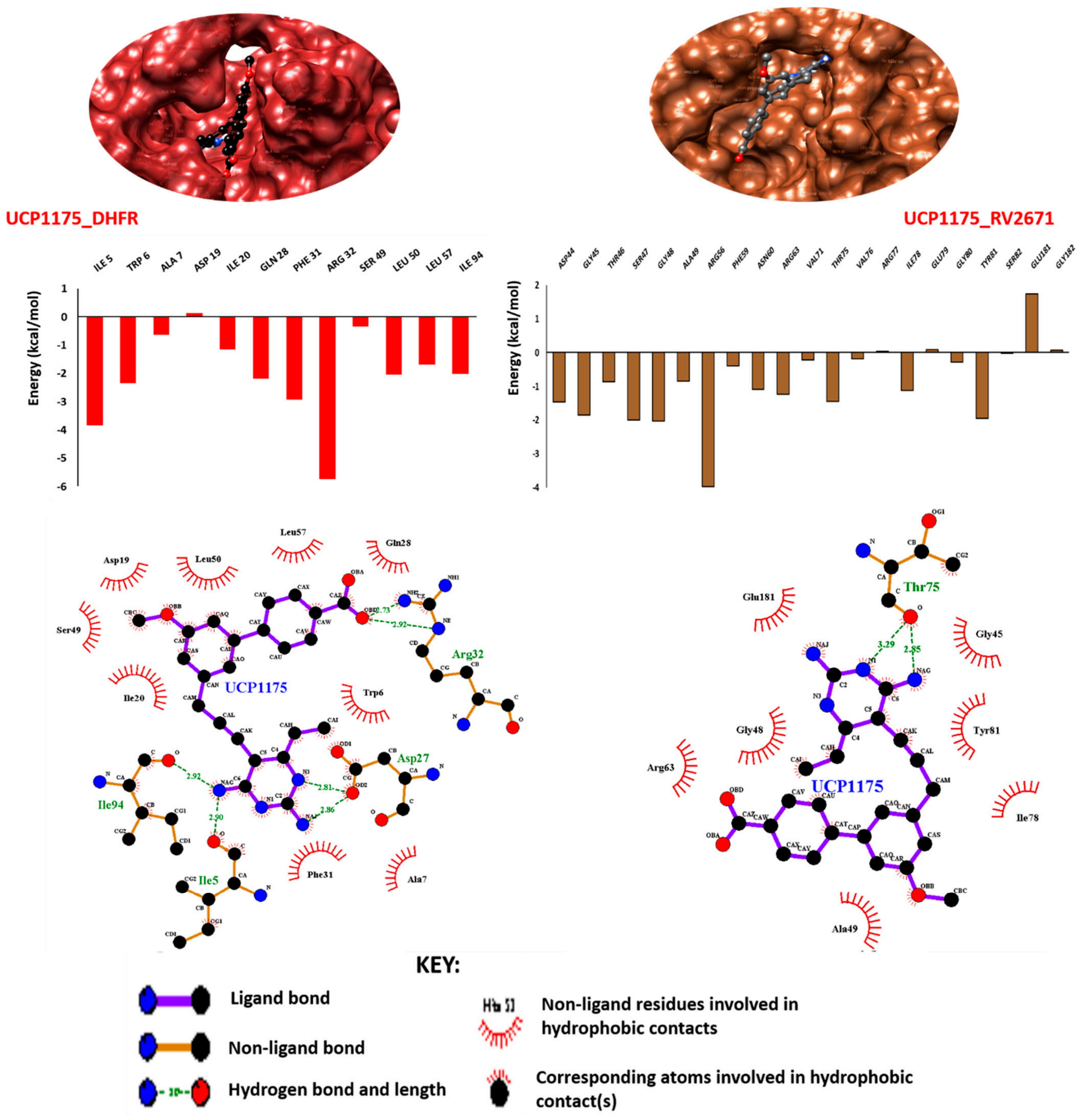

Figure S4: Binding landscape and molecular interaction plots of the DHFR and RV2671 in complex with UCP1175. Graphs demonstrated the energy contributions of each active-site residue to the final free-binding energy of each system.

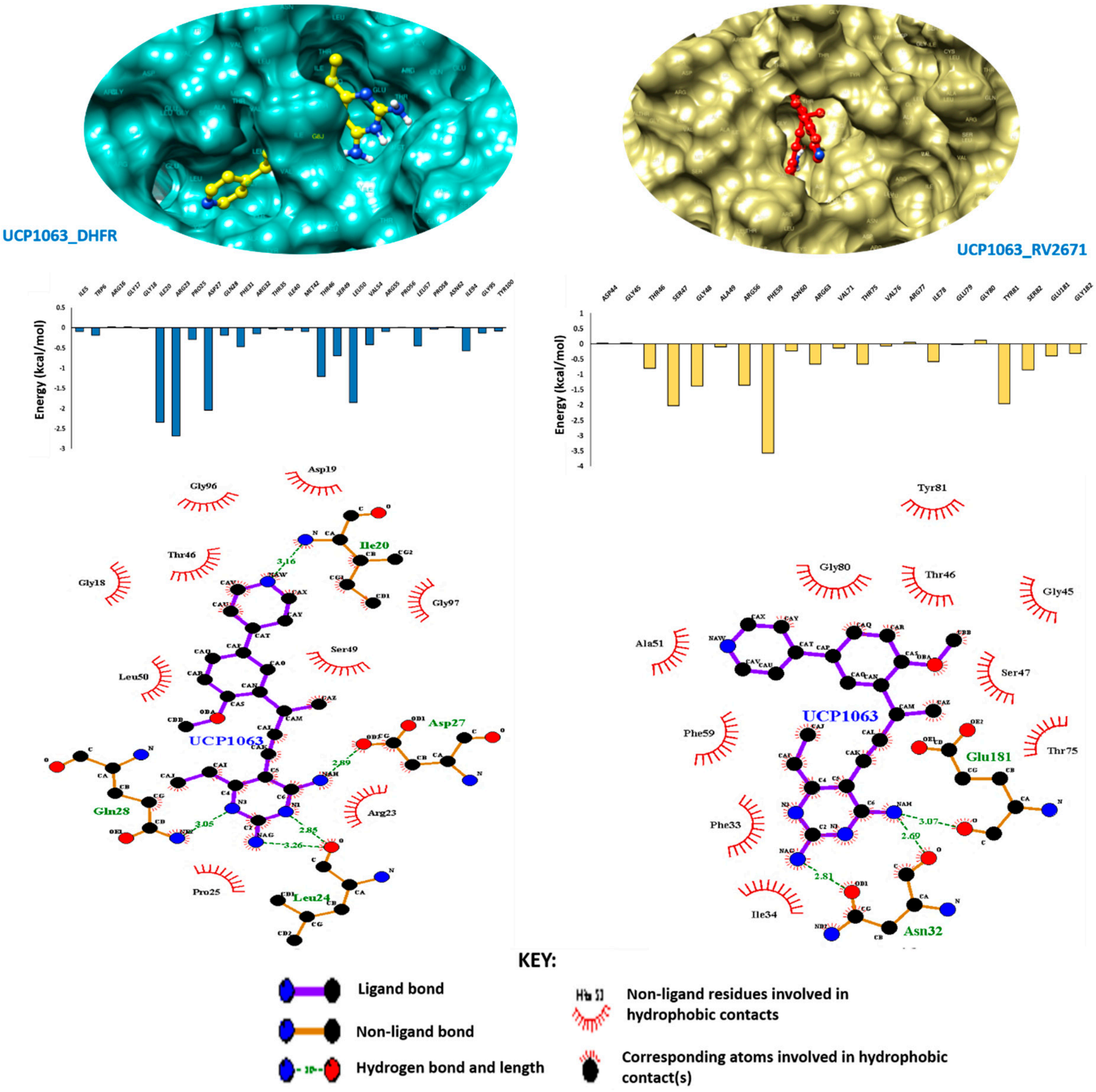

Figure S5: Binding landscape and molecular interaction plots of the DHFR and RV2671 in complex with UCP1063. Graphs demonstrated the energy contributions of each active-site residue to the final free-binding energy of each system
